# A comparison of dispersing media for various engineered carbon nanoparticles

**DOI:** 10.1186/1743-8977-4-6

**Published:** 2007-07-27

**Authors:** Mary C Buford, Raymond F Hamilton, Andrij Holian

**Affiliations:** 1 Center for Environmental Health Sciences, Department of Biomedical and Pharmaceutical Sciences, University of Montana, Missoula, Montana, USA

## Abstract

**Background:**

With the increased manufacture and use of carbon nanoparticles (CNP) there has been increasing concern about the potential toxicity of fugitive CNP in the workplace and ambient environment. To address this matter a number of investigators have conducted *in vitro *and *in vivo *toxicity assessments. However, a variety of different approaches for suspension of these particles (culture media, Tween 80, dimethyl sulfoxide, phosphate-buffered saline, fetal calf serum, and others), and different sources of materials have generated potentially conflicting outcomes. The quality of the dispersion of nanoparticles is very dependent on the medium used to suspend them, and this then will most likely affect the biological outcomes.

**Results:**

In this work, the distributions of different CNP (sources and types) have been characterized in various media. Furthermore, the outcome of instilling the different agglomerates, or size distributions, was examined in mouse lungs after one and seven days. Our results demonstrated that CNP suspended in serum produced particle suspensions with the fewest large agglomerates, and the most uniform distribution in mouse lungs. In addition, no apparent clearance of instilled CNP took place from lungs even after seven days.

**Conclusion:**

This work demonstrates that CNP agglomerates are present in all dispersing vehicles to some degree. The vehicle that contains some protein, lipid or protein/lipid component disperses the CNP best, producing fewer large CNP agglomerates. In contrast, vehicles absent of lipid and protein produce the largest CNP agglomerates. The source of the CNP is also a factor in the degree of particle agglomeration within the same vehicle.

## Background

In the last decade there has been a dramatic increase in research and technology at the nanometer scale. Carbon nanoparticles (CNP) are an important component of this nanotechnology revolution due to the unique electrical, physical and thermal qualities of these particles [1]. CNP exist in three primary forms – fullerene carbon spheres (C60CS), single-walled nanotubes (SWNT) and multi-walled nanotubes (MWNT). Another variant of the MWNT is the double-walled nanotube, which will not be addressed in this study. These particles are generated through a variety of methods including electrical arc discharge, laser vaporization, chemical-vapor deposition (CVD), and high pressure carbon monoxide (HiPco) [2]. All of these production methods produce some metal contaminants, typically Fe, Ni, Y, and Co [2].

Previous studies have reported CNP toxicity in mouse and rat models [3-5], but there is little known about the mechanism of CNP toxicity. The unique physical characteristics of these particles present a new class of material because of their size and extreme hydrophobic nature. This study focuses specifically on the characterization of CNP in regards to particle source and methods of dispersion for biomedical studies. Dispersion studies have been done on SWNT using various surfactants and polymers [6], but the results were not translatable to biological research because the vehicles described would be toxic to most biological systems.

*In vitro *and *in vivo *toxicology studies have used a variety of strategies regarding CNP dispersion into a biological vehicle. CNP vehicles have included cell culture media [7-9], with and without fetal calf serum (FCS), pluronic surfactant [10], mouse serum [3], fetal calf serum (FCS) [11], 1% Tween 80 in phosphate buffered saline (PBS) [4,12], PBS alone [5], dimethyl sufoxide (DMSO) [13], Tyrode's solution [14], and tetrahydrofuran (THF) solvent to create water-soluble fullerenes [15]. Another variable with regard to these studies is the variety of sources for similar particle types of CNP produced by different methods containing different contaminants. The end result has been a difference in findings regarding the cytotoxicty and mechanism of action of various CNP in biological models.

The purpose of this study is to give researchers a frame of reference with regard to CNP agglomeration in a variety of media. The hypothesis of this work is that all dispersing media (at least the one's used to date) produce some degree of CNP agglomeration. In addition, optimal dispersion of CNP in a biological system must include some lipid and/or protein component. The relative dispersion characteristics of any given CNP must be determined empirically because CNP from different sources have variable dispersion characteristics in the same media.

## Results

### Relative dispersal of CNP in various media vehicles

**Figure 1:**
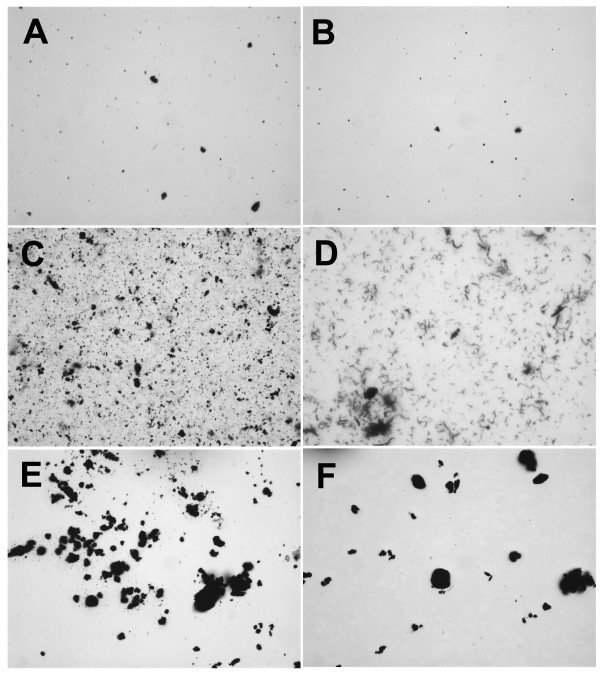
**Carbon nanoparticles suspended in 100% fetal calf serum (FCS)**. **A**) C60CS from SES Research, **B**) C60CS from BuckyUSA, **C**) SWNT from SES Research, **D**) SWNT from CNI, **E**) MWNT from SES Research, and **F**) MWNT from NanoLab. All particle suspensions were at 5 mg/ml. Magnification – 400×.

In order to compare agglomeration properties of CNP, various suspension media vehicles were selected based on the types used in previous publications. CNP were suspended at 5 mg/ml and 10 μl samples were analyzed by light microscopy at 400× magnification to examine relative CNP agglomeration states. Figures 1 thru 7 are organized whereby each figure represents a suspension media. The progression from Figure 1 to Figure 7 represents the best CNP dispersal media to the worst CNP dispersal media. CNP types from different sources are placed side-by-side for reference (e.g., A compared to B, C compared to D, and E compared to F). Figures A, C and E are CNP from SES Research and B, D and F are CNP from alternative sources (described below).

**Figure 2:**
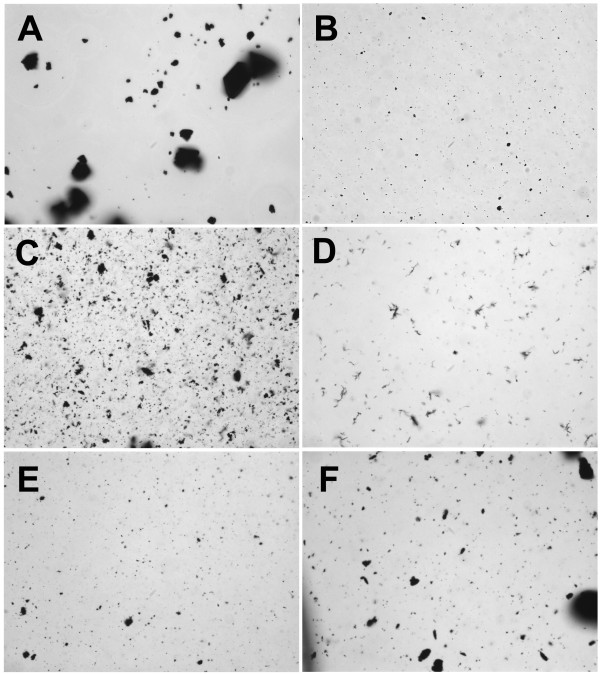
**Carbon nanoparticles suspended in 7.5% bovine serum albumin (BSA) in phosphate buffered saline (PBS)**. **A**) C60CS from SES Research, **B**) C60CS from BuckyUSA, **C**) SWNT from SES Research, **D**) SWNT from CNI, **E**) MWNT from SES Research, and **F**) MWNT from NanoLab. All particle suspensions were at 5 mg/ml. Magnification – 400×.

**Figure 3:**
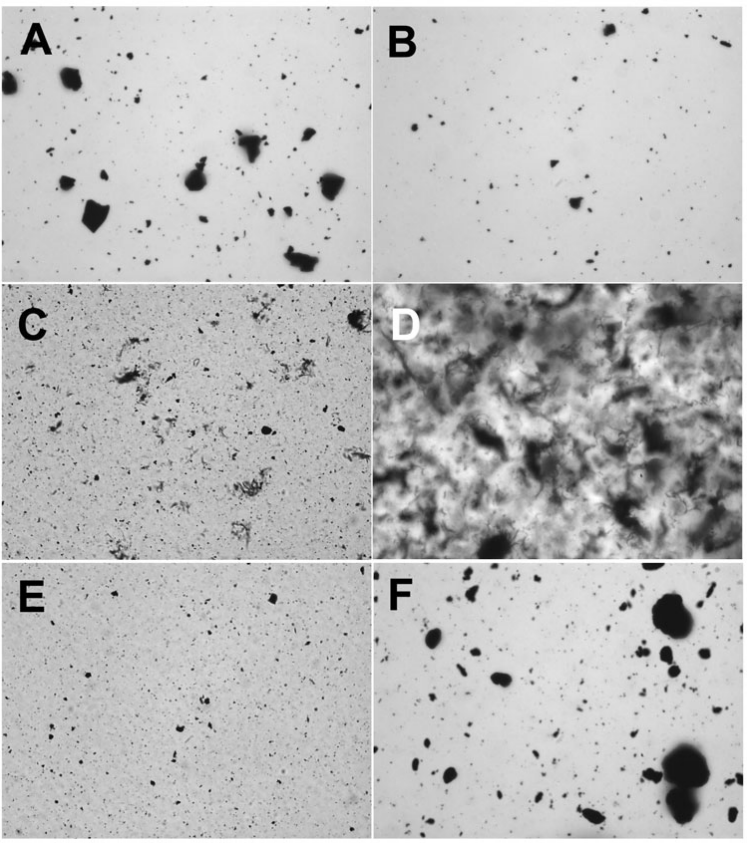
**Carbon nanoparticles suspended in RPMI media with 10% fetal calf serum (FCS)**. **A**) C60CS from SES Research, **B**) C60CS from BuckyUSA, **C**) SWNT from SES Research, **D**) SWNT from CNI, **E**) MWNT from SES Research, and **F**) MWNT from NanoLab. All particle suspensions were at 5 mg/ml. Magnification – 400×.

**Figure 4:**
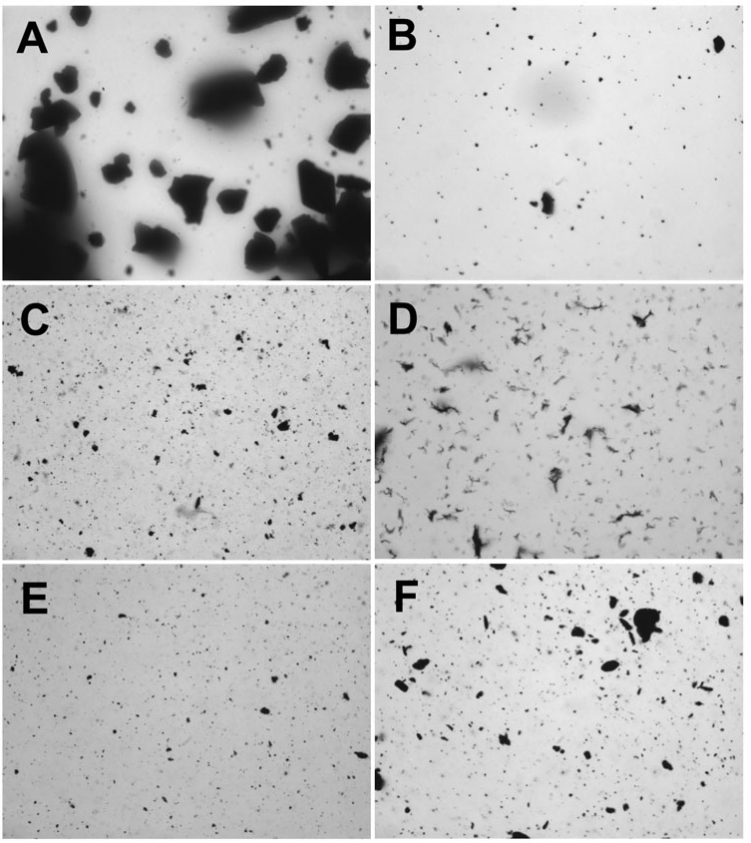
**Carbon nanoparticles suspended in 100% delipidated fetal calf serum (delipFCS)**. **A**) C60CS from SES Research, **B**) C60CS from BuckyUSA, **C**) SWNT from SES Research, **D**) SWNT from CNI, **E**) MWNT from SES Research, and **F**) MWNT from NanoLab. All particle suspensions were at 5 mg/ml. Magnification – 400×.

**Figure 5:**
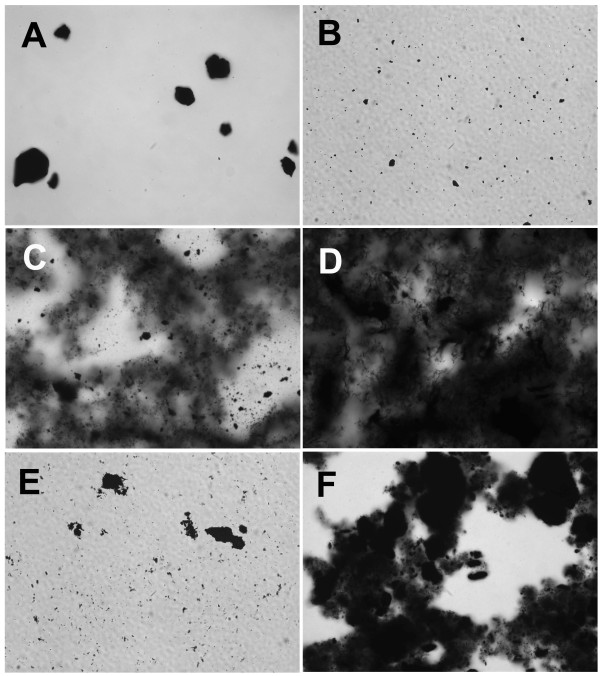
**Carbon nanoparticles suspended in 1% tween 80 in phosphate buffer saline (PBS)**. **A**) C60CS from SES Research, **B**) C60CS from BuckyUSA, **C**) SWNT from SES Research, **D**) SWNT from CNI, **E**) MWNT from SES Research, and **F**) MWNT from NanoLab. All particle suspensions were at 5 mg/ml. Magnification – 400×.

**Figure 6:**
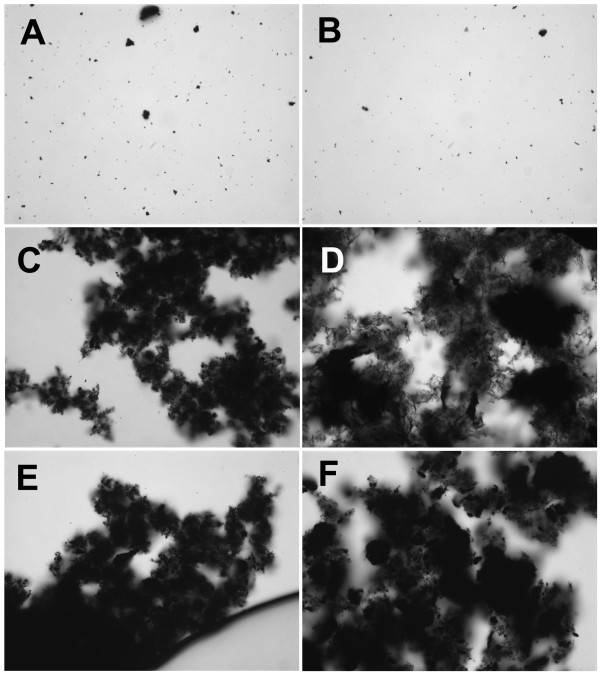
**Carbon nanoparticles suspended in phosphate buffered saline (PBS)**. **A**) C60CS from SES Research, **B**) C60CS from BuckyUSA, **C**) SWNT from SES Research, **D**) SWNT from CNI, **E**) MWNT from SES Research, and **F**) MWNT from NanoLab. All particle suspensions were at 5 mg/ml. Magnification – 400×.

**Figure 7:**
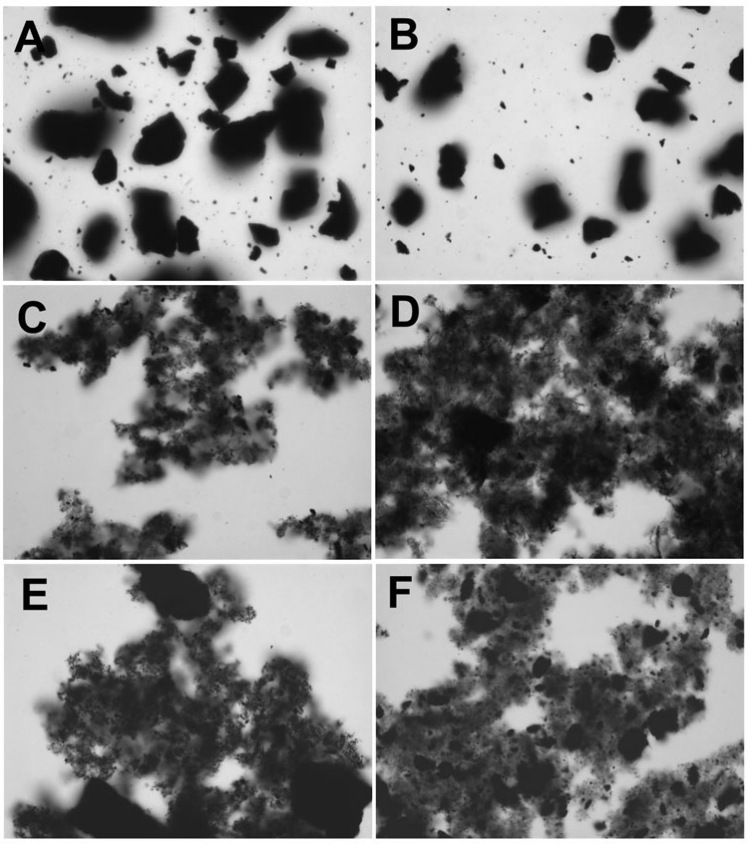
**Carbon nanoparticles suspended in 100% dimethyl sulfoxide (DMSO)**. **A**) C60CS from SES Research, **B**) C60CS from BuckyUSA, **C**) SWNT from SES Research, **D**) SWNT from CNI, **E**) MWNT from SES Research, and **F**) MWNT from NanoLab. All particle suspensions were at 5 mg/ml. Magnification – 400×.

Descriptive data (median size area and maximum size area) on all dispersed CNP can be found in Table 1. These data were obtained by ImagePro software as described in Methods. Vehicles that produced mass agglomeration (e.g., SWNT and MWNT in 1% tween 80 and DMSO), were omitted from the table and analyses. All images that could be analyzed for particle area produced similar exponential histograms with a large number of smaller agglomerates and a small number of large agglomerates (with some being very large relative to the median agglomerate area). A larger median area indicates the presence of more frequent large agglomerates, whereas the maximum area is the area for the largest single agglomerate analyzed indicating the most extreme agglomerate state for a particular CNP in a particular media. Taken together, the results in Table 1 indicate that the median areas for all suspended agglomerates are relatively consistent regardless of particle type or vehicle. In contrast, the area of the largest observed agglomerate is extremely variable depending on particle type, particle source and vehicle used.

**Table 1:** Agglomerate carbon nanoparticle counts, median area, and maximum area

**Veh: FCS**	**median area (μm^**2**^)**	**max area (μm^**2**^)**
Crude Fullerenes	.003	.878
C60CS (SES)	.003	.157
C60CS (BUSA)	.003	.076
SWNT (SES)	.003	.333
SWNT (CNI)	.006	5.915
MWNT (SES)	.005	2.851
MWNT (NanoLab)	.058	1.462
		

**Veh: 7.5% BSA**	**median area (μm^**2**^)**	**max area (μm^**2**^)**

Crude Fullerenes	.004	.478
C60CS (SES)	.024	2.813
C60CS (BUSA)	.001	.034
SWNT (SES)	.003	.378
SWNT (CNI)	.005	.181
MWNT (SES)	.002	.127
MWNT (NanoLab)	.004	2.344
		

**Veh: RPMI + FCS**	**median area (μm^**2**^)**	**max area (μm^**2**^)**

Crude Fullerenes	.004	2.45
C60CS (SES)	.004	.842
C60CS (BUSA)	.003	.208
SWNT (SES)	.002	.434
SWNT (CNI)	.005	16.689
MWNT (SES)	.002	.093
MWNT (NanoLab)	.014	3.825
		

**Veh: FCS (delipid)**	**median area (μm^**2**^)**	**max area (μm**^2^**)**

Crude Fullerenes	.004	2.739
C60CS (SES)	.127	14.031
C60CS (BUSA)	.004	.328
SWNT (SES)	.003	.133
SWNT (CNI)	.006	.704
MWNT (SES)	.002	.105
MWNT (NanoLab)	.003	.826
		

**Veh: 1% Tween 80**	**median area (μm^**2**^)**	**max area (μm^**2**^)**

Crude Fullerenes	.003	.995
C60CS (SES)	.002	2.379
C60CS (BUSA)	.003	.068
SWNT (SES)	Lg. agglomerates	Lg. agglomerates
SWNT (CNI)	Lg. agglomerates	Lg. agglomerates
MWNT (SES)	.002	.869
MWNT (NanoLab)	Lg. agglomerates	Lg. agglomerates
		

**Veh: PBS**	**median area (μm^**2**^)**	**max area (μm^**2**^)**

Crude Fullerenes	.005	1.567
C60CS (SES)	.003	.615
C60CS (BUSA)	.003	.096
SWNT (SES)	Lg. agglomerates	Lg. agglomerates
SWNT (CNI)	Lg. agglomerates	Lg. agglomerates
MWNT (SES)	Lg. agglomerates	Lg. agglomerates
MWNT (NanoLab)	Lg. agglomerates	Lg. agglomerates
		

**Veh: DMSO**	**median area (μm^**2**^)**	**max area (μm^**2**^)**

Crude Fullerenes	.003	.849
C60CS (SES)	.008	12.333
C60CS (BUSA)	.005	3.90
SWNT (SES)	Lg. agglomerates	Lg. agglomerates
SWNT (CNI)	Lg. agglomerates	Lg. agglomerates
MWNT (SES)	Lg. agglomerates	Lg. agglomerates
MWNT (NanoLab)	Lg. agglomerates	Lg. agglomerates

CNP suspended in 100% FCS are shown in Figure 1. C60CS dispersed well with only a few visible large agglomerates (Figures 1A and 1B). SWNT (Figures 1C and 1D) dispersed with uniform small CNP agglomerates. The only visible difference between the SWNT agglomeration states is that the SWNT from SES agglomerated in clumps, whereas the SWNT from CNI agglomerated in fibre-like stands. The MWNT appeared to agglomerate in uniform small to medium sized clumps regardless of source (Figures 1E and 1F).

CNP suspended in 7.5% BSA/PBS are shown in Figure 2. There was a significant difference in how the C60CS dispersed in this media with the SES C60CS forming large and small agglomerates (Figure 2A), with the C60CS from BuckyUSA forming only few visible small agglomerates (Figure 2B). The SWNT appeared to disperse in a similar manner to what was described in Figures 1C and 1D, with the formation of uniform small agglomerates (Figure 2C and 2D). The MWNT appeared to disperse better in this media compared to FCS with the formation of small visible agglomerates (Figures 2E and 2F). However, larger agglomerates appeared in the MWNT from NanoLab (Figure 2F).

CNP suspended in RPMI media with 10% FCS are shown in Figure 3. With regard to C60CS, this vehicle was very similar to the BSA/PBS with large agglomerates only appearing in the SES C60CS sample (Figure 3A), although larger agglomerates were also present in the BuckyUSA C60CS sample (Figure 3B). This is possibly the best dispersal media for SWNT and MWNT from SES as only small agglomerates were visible (Figure 3C and 3E). In contrast, SWNT from CNI became a swirling mass agglomerate (Figure 3D), and MWNT from NanoLab formed very large agglomerates (Figure 3F). This figure illustrates how CNP from different sources can be completely different with regard for formation of agglomerates in a particular vehicle.

CNP suspended in 100% delipidated FCS are shown in Figure 4. This vehicle appears to be a good dispersing media for all CNP with the exception of C60CS from SES where very large agglomerates formed (Figure 4A), and MWNT from NanoLab with the formation of some larger agglomerates (Figure 4F). The other CNP only created small agglomerates in this vehicle.

Figure 5 illustrates the CNP suspended in 1% tween 80 in PBS. This represents the first of the generally poor dispersal vehicles. Large agglomerates appeared with C60 from SES (Figure 5A). In contrast, this vehicle dispersed well for C60CS from BuckyUSA (Figure 5B). The SWNT from SES formed a cloud of agglomerated CNP with some more solid agglomerates visible (Figure 5C). The SWNT from CNI formed a swirling massive agglomeration of CNP (Figure 5D). MWNT from SES dispersed relatively well in the tween 80 vehicle (Figure 5E), whereas the MWNT from NanoLab agglomerated similarly to the SWNT samples described above (Figure 5F).

The results shown in Figure 6 represent another poor dispersal vehicle, PBS alone. The PBS vehicle was ineffective in dispersing both SWNT and MWNT regardless of source, as massive CNP clumps were apparent (Figure 6C–F). In contrast, the C60CS from both sources appeared to be relatively dispersed (Figures 6A and 6B).

The worst dispersal vehicle (100% DMSO) is shown in Figure 7. All CNP tested formed large agglomerates in this vehicle. The C60CS were characterized by large solid clumps (Figure 7A and 7B), and the other CNP formed large loose clusters of agglomerated nanoparticles.

### Control particle in the various dispersal media

**Figure 8:**
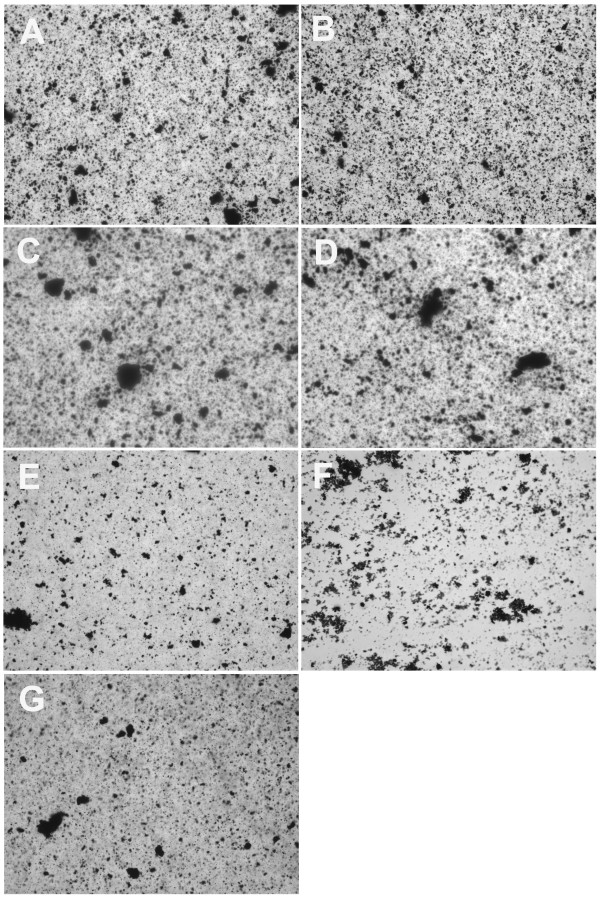
**Crude carbon fullerenes suspended in various media vehicles**. **A**) 100% fetal calf serum (FCS), **B**) 7.5% bovine serum albumin (BSA) in phosphate buffered saline (PBS), **C**) RPMI media with 10% fetal calf serum (FCS), **D**) 100% delipidated fetal calf serum (delipFCS), **E**) 1% tween 80 in phosphate buffer saline (PBS), **F**) phosphate buffered saline (PBS), and **G**) 100% dimethyl sulfoxide (DMSO). All particle suspensions were at 5 mg/ml. Magnification – 400×.

Figure 8 represents the control particle, which is a crude fullerene carbon ash dispersed in all 7 of the vehicles tested. All of the vehicles produced a similar pattern of dispersal with very small to medium sized agglomerates formed. The number of agglomerates was larger in these samples due to the density of the particle compared to CNP. This figure illustrates that the differences seen in the CNP suspended in various media above were due to structural/physical properties in the specific CNP particular to the nano scale. Crude carbon particles not on the nano scale do not react to the differences in the vehicle makeup.

### Effect of using different dispersal vehicles in SWNT lung instillations in a mouse model

**Figure 9:**
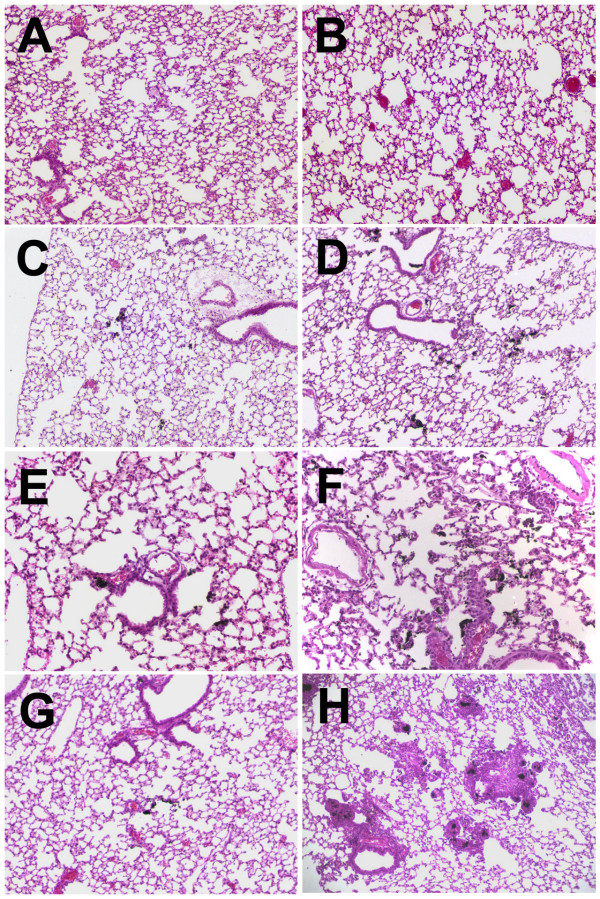
Representative photomicrographs of SWNT deposition in mouse lungs after 24 hours (A thru F) and 7 days (G and H). **A**) Lung morphology following PBS vehicle control instillation, **B**) Lung morphology following 100% FCS vehicle control instillation, **C**) Lung morphology following 250 μg SWNT instillation in PBS vehicle **D**) Lung morphology following 250 μg SWNT instillation in 100% FCS vehicle, **E**) Lung morphology following 250 μg SWNT instillation in PBS vehicle (high mag.), **F**) Lung morphology following 250 μg SWNT instillation in 100% FCS vehicle (high mag.), **G**) Lung morphology 7 days following 250 μg SWNT instillation in PBS vehicle **H**) Lung morphology 7 days following 250 μg SWNT instillation in 100% FCS vehicle Magnification – 100× except for E and F at 200×.

In order to determine the effect of the CNP vehicle on lung dispersion during particle instillation, BALB/c mice were given 250 μg instillations in either 100% FCS or PBS (representing the two extreme agglomerate states of the SWNT). After 24 hours the lungs showed significant differences in SWNT deposition as shown in Figure 9. Figure 9A (PBS) and Figure 9B (100% FCS) represent the respective vehicle controls at 100× magnification. The comparative SWNT dispersion patterns can be found in Figure 9C and Figure 9D, respectively. The blackened areas represent the accumulation of SWNT which is more generally dispersed in Figure 9D which used the 100% FCS vehicle. This observation was more pronounced at higher magnifications (200×), as illustrated in Figures 9E and 9F. Lung histology prepared 7 days post-CNP instillation showed only trace amounts of SWNT in the lungs using the PBS vehicle (Figure 9G). In contrast, the SWNT instilled with 100% FCS had persisted in the lung tissue and produced areas of increased cellularity indicative of sustained inflammation (Figure 9H).

## Discussion

Oberdorster et.al., suggested that CNP toxicity could be dependent on particle size, size distribution, agglomeration state, shape, surface chemistry, surface area, and surface charge [1]. All of these qualities can be affected directly, or indirectly, by the suspension media makeup. Researchers should be aware of how CNP toxicity could be potentially modified by the choice of vehicle or source of material in a study. Due to the lack of standardization in CNP research, results may seem contradictory when compared. For example, using the same CNP types and similar suspension media Jia et.al., found *in vitro *toxicity [8], whereas Hamilton et.al., found very little CNP cytotoxicity *in vitro *[11]. The only variable that could account for the difference was the different sources of the CNP. Therefore, contaminants could account for the toxicity reported for some CNP.

Alternative media for CNP dispersion, mouse bronchoalveolar lavage fluid (BALF) and lung surfactant component dipalmitoyl phosphatidylcholine (DPPC), not included in this study can be found in Sager et.al.,[16]. Their findings were consistent with ours in that lipid-protein mixture (BALF or PBS containing protein and DPPC) was the best dispersant. Use of BALF as a dispersing media for CNP, although effective, creates problems with reproducibility (unknown dilution factor) from experiment to experiment. On the other hand, synthetic lung surfactant may be a viable alternative, especially for in vivo experiments. Commercial sources of synthetic lung surfactant can be cost prohibitive, however simple components of surfactant can be obtained and used for CNP dispersion. Caution should be exercised in the use of lung surfactant or its components as a dispersant vehicle due to evidence that these vehicles can modify the toxicity of other respirable particles (diesel soot and silicate dusts) *in vivo *and *in vitro *by surface absorption, altering the surface chemistry of the particle [17]. A similar effect could occur with CNP. The results from the current study suggest that some protein component should be considered in the mixture regardless of the vehicle choice.

With regard to *in vivo *particle instillations, we have demonstrated that CNP dispersion is critical to effective distribution throughout the lung. Several earlier studies used PBS and 1% Tween 80 as the CNP vehicle [4,5,12]. Based on our findings, the CNP was probably extremely agglomerated to the point that it was not distributed evenly in the lungs. In fact, 2 of the 3 studies reported respiratory blockage and animal deaths resulting from CNP instillation [3,4,12]. This probably could have been avoided if other vehicle options were examined. In contrast, in vivo studies that used serum as the CNP vehicle [3,11], did not report these problems (note: Lam et.al., did have to adjust the CNP amount instilled from the original lethal 1 mg bolus dose) and probably achieved even distribution throughout the mouse lungs.

## Conclusion

Complete or total dispersion of CNP is not practical in a biological model. Toxicology studies using a biological system will be evaluating the CNP with some degree of agglomeration. The relative CNP agglomeration is determined by two factors – the stock suspension vehicle and the source of the material. This study has demonstrated that the same type of CNP from different sources can behave differently in the same dispersing media. In addition, SWNT and MWNT are more prone to agglomeration than C60CS. The most important factor in how well a CNP disperses is the presence of protein, lipid or protein/lipid combination in the suspending vehicle. This finding is consistent with Sager et.al. [16] where lipid-protein was necessary for ultrafine carbon black and ultrafine titanium dioxide dispersion. The absence of lipid or protein in the vehicle results in relatively large CNP agglomerates, with protein being the more critical of the two.

The dispersion of agglomerate CNP is crucial to effective deposition in the lung for *in vivo *studies. The establishment of standard reference materials for CNP, and a standard dispersion protocol would be of great benefit to toxicology studies, because it would allow researchers to achieve some degree of concordance. In the absence of standard reference materials, it is important for all CNP researchers to be aware that the dispersion characteristics of any given CNP can be optimized by experimenting with different vehicles, and that no one vehicle is optimal for all CNP types or sources. This can only be established by experimental observation.

This study observed CNP agglomeration on the light microscopic scale. Although it would have been possible to have further refined the analysis to higher resolution, the current study using light microscopic analysis was sufficient to demonstrate a wide variety of agglomeration states using different dispersion media and CNP from different sources. This study was intended to serve as a frame of reference for researchers interested in using CNP for biological studies. We are not suggesting that one dispersion media is superior to another, simply that they are different. This study does not address how various dispersion media can alter CNP biological activity. It is up to each researcher to determine the effect of dispersing media on a particular CNP in their specific model system.

## Methods

### Particles

Several CNP were used for these studies. The particles used and their respective sources are listed as follows: C60CS from SES Research (Houston, TX), C60CS from BuckyUSA (Houston, TX), SWNT from SES Research (Houston TX), SWNT from Carbon Nanotechnologies Inc. (CNI, Houston, TX), MWNT from SES Research (Houston, TX), MWNT from Nanolab (Newton, MA), and crude fullerene (elemental carbon not on the nanoscale) was used as a control particle (generous donation from Maria Morandi). Detailed CNP characterization can be found in Table 2.

**Table 2:** Carbon nanoparticle characterization

**Source**	**Particle**	**MMethod**	**Purity**	**Contaminants**	**Diameter**	**Length**
BuckyUSA	C60CS	C plasma	99.9%	N/A	0.7 nm	N/A
SES	C60CS	C plasma	99.9%	N/A	10.18 Å	N/A
CNI	SWNT	HiPco	85%	C ash, Fe	0.8 – 1.2 nm	0.1–1 μm
SES	SWNT	CVD	75%	C, Ni, Y	< 2 nm	1–5 μm
NanoLab	MWNT	CVD	95%	C, Fe, Co, Ni	30 ± 15 nm	1–5 μm
SES	MWNT	CVD	75%	C, Ni, Y	10 – 30 nm	1–2 μm

### Suspension Media

Various suspension media were used in these studies in order to demonstrate the hydrophobic characteristics of CNP, and the potential need for a lipid interaction, causing better particle dispersion. Seven media were used in total and they were as follows: cell culture media RPMI (HEPES buffered w/L-glutamine, MediaTech) with 10% fetal calf serum (FCS), FCS alone (Hyclone), phosphate buffered saline (PBS, Diamedix), dimethyl sulfoxide (DMSO, stored under argon in amber vials, Sigma, St. Louis, MO), 1%Tween80 (Sigma, St. Louis, MO) in PBS, delipidated FCS (Biomeda Corporation), and 7.5% bovine serum albumin (BSA)/PBS solution (Sigma, St. Louis, MO). All particle suspensions were freshly prepared before instillation by suspension in sterile media and dispersed by sonication (Sonicator Ultrasonic Processor – Misonix, Farmingdale, NY) for one minute and vortexed for 10 sec before sonication and prior to mounting onto microscope slide.

### Imaging

The various stock CNP suspensions were made at a concentration of 5 mg/ml and 10 μl was pipetted onto a pre-cleaned and charged microscope slide, and cover-slipped. They were photographed at 400× under white light using Nuance Imaging software. In order to determine particle size and count for frequency distribution analysis, ImagePro software was employed to determine particle frequency and area. The threshold sensitivity of the imaging was set to include all visible particles. The area for each particle was calculated in number of pixels. This pixel count was then converted to square microns. One square micron was equal to 12544 pixels. Particle area was calculated as number of pixels per particle divided by 12544 equalling square microns of particle area.

### Animals

Balb/c mice (Jackson Laboratories, Bar Harbor, ME) were used for all of the *in vivo *studies. All mice were used at 12 weeks of age. Animals were housed in microisolators on a 12:12 h light-dark cycle. The mice were maintained on an OVA-free diet and given deionized water ad *libitum*. Euthanasia was performed by intraperitoneal injection of a lethal dose of sodium pentobarbital. All animal procedures were approved by the University of Montana Institutional Animal Care and Use Committee.

### Particle Instillation

All CNP exposures were administered intratracheally at a dose of 250 μg per mouse. Mice were anesthetized using 100 mg/kg Ketamine and 5 mg/kg Xylazine via IP injection. Mice were checked after a few minutes to verify that the anesthetic agents were effective. The mouse was then placed on its back and feet secured with tape. Hair was shaved from the upper thorax area and ethanol was applied. An incision, approximately 1 cm in length was made using a sterile blade in upper thorax area. Salivary glands were pushed aside to expose the trachea. Particulates were administered by injection into the trachea using a 23G sterile needle. The incision was closed with VetBond adhesive. The mice were monitored until they fully recovered from the procedure and then returned to the animal care facility. The mice were left for one or seven days before removal of the lungs for histological analysis.

### Histology

Mice were euthanized by intraperitoneal injection of a lethal dose of sodium pentobarbital. The whole lungs were removed are fixed in 4% paraformaldehyde overnight. They were rinsed in a phosphate saline buffer (PBS) and processed for paraffin embedding. Sections were cut at 5 μm and mounted onto charged microscope slides. They were stained with hematoxylin and eosin (basic H&E stain) and analysed under white light at various magnifications.

### Statistical Analysis

Nonparametric descriptive statistics including median area, and maximum area were used to describe the particle distributions. When particle distributions could be determined the distribution had an exponential curve with the frequency of smaller particle areas much greater than the frequency of larger particle areas resulting in the exponential fall off in frequency over increasing area.

## Competing interests

The author(s) declare that they have no competing interests.

## Authors' contributions

MCB and RFH designed, conducted the study and prepared the text for the manuscript. AH provided overall direction for the study and edited the final text. All authors have read and approved the final manuscript.
